# Supplementary material for: Ceramide Phosphoethanolamine as a Possible Marker of Periodontal Disease
Source: Membranes (Basel). 2022 Jun 25;12(7):655. doi: 10.3390/membranes12070655 (PMC9324278; doi:10.3390/membranes12070655)
Supplement: Supplementary file 1 [file membranes-12-00655-s001.zip › membranes-1771813-supplementary.pdf]

Supplementary

Table S1: Transition masses, dwell time and collision energies of multiple reaction monitoring (MRM) measurements of *T. forsythia* lipid sample.

MRM transitions CPE series

Q1 Mass (amu)	Q3 Mass (amu)	Dwell (msec)	Collision energy (V)
605.7	464.5	150	31
631.7	490.5	150	31
633.7	492.5	150	31
635.7	494.5	150	31
647.7	506.5	150	31
649.7	508.5	150	31
659.7	518.5	150	31
661.7	520.5	150	31
663.7	522.5	150	31
675.7	534.5	150	31
677.7	536.5	150	31
687.7	546.5	150	31
689.7	548.5	150	31
691.7	550.5	150	31
703.7	562.5	150	31
717.7	576.5	150	31

MRM transitions 2'-OH-DH-CPE series

Q1 Mass (amu)	Q3 Mass (amu)	Dwell (msec)	Collision energy (V)
637.7	496.5	200	31
651.7	510.5	200	31
665.7	524.5	200	31
679.7	538.5	200	31
693.7	552.5	200	31
707.7	566.5	200	31
721.7	580.5	200	31

Table S2: Mass spectrometer MRM specific parameters.

Triple quadrupole MS (SCIEX API 3000) parameters	
Scan Type	MRM
Polarity	Positive
Scan Mode	N/A
Ion Source	Turbo Spray
Resolution Q1	Unit
Resolution Q3	Unit
Intensity Threshold	0.00 cps
Settling Time	0.0000 msec
MR Pause	5.0070 msec

MCA	No
Step Size	0.00 amu
NEB	8
CUR	8
IS	5000
TEM	300
CAD	4
DP	60
FP	250
EP	10
CE	31
CXP	20

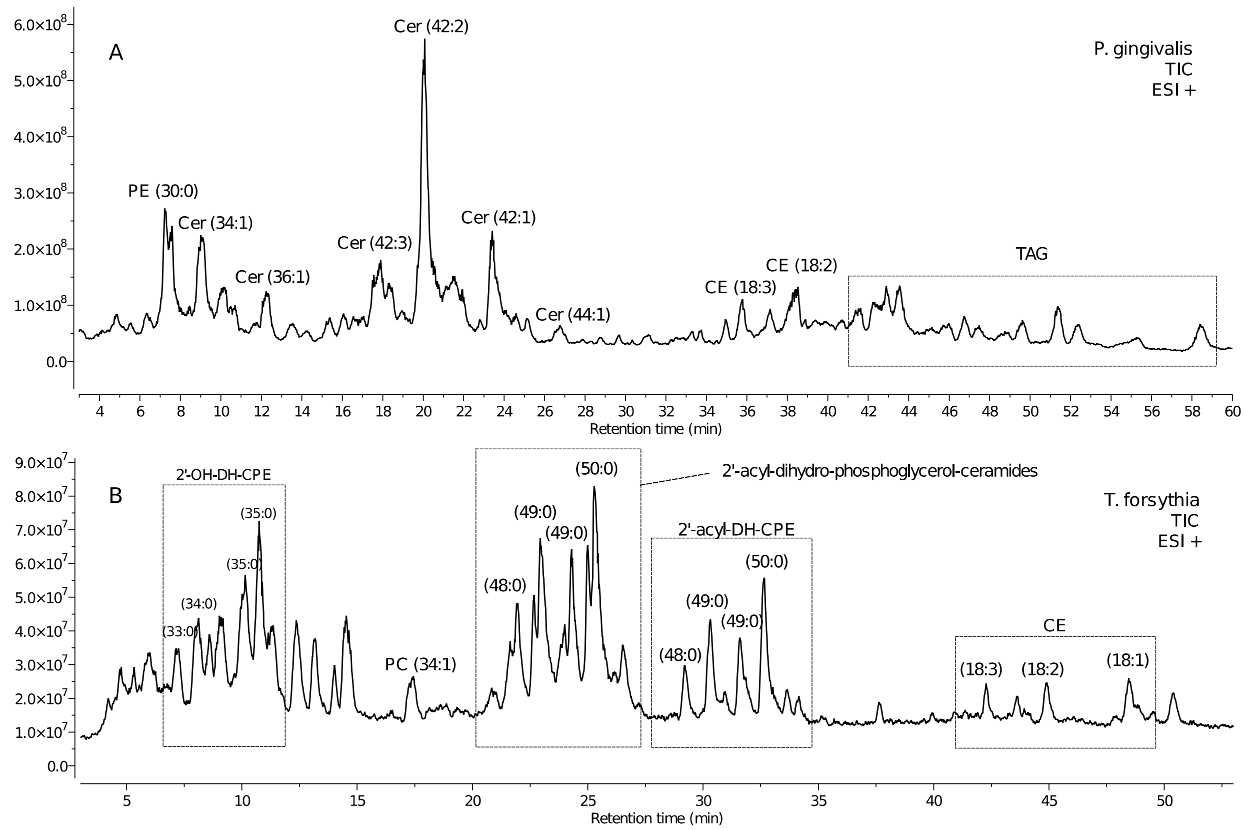

Figure S1: Total ion current (TIC) chromatograms of lipid extract of *P. gingivalis* (A) and *T. forsythia* (B). Main peaks are labelled with the corresponding, identified lipid. Note that *T. forsythia* chromatogram (B) presents 2'-acyl-dihydro-ceramides with phosphoglycerol head along with 2′-OH-DH- and 2′-acyl-DH- CPE.
